# Excited states of mono- and biruthenium(II) complexes adsorbed on nanocrystalline titanium dioxide studied by electroabsorption spectroscopy

**DOI:** 10.1038/s41598-024-81957-z

**Published:** 2025-02-07

**Authors:** Daniel Pelczarski, Błażej Gierczyk, Maciej Zalas, Malgorzata Makowska-Janusik, Waldemar Stampor

**Affiliations:** 1 Department of Molecular Photophysics, Institute of Applied Physics and Mathematics, Gdańsk University of Technology, Narutowicza 11/12, 80-233 Gdańsk, Poland; 2 Faculty of Chemistry, Adam Mickiewicz University, Poznań, Uniwersytetu Poznańskiego 8, 61-614 Poznań, Poland; 3 Faculty of Science and Technology, Jan Dlugosz University, Al. Armii Krajowej 13/15, 42-200 Czestochowa, Poland

**Keywords:** Electronic structure, Excited states, Surfaces, interfaces and thin films, Optical spectroscopy, Solar cells

## Abstract

Comprehensive characterization of the lowest energy electronic excited states for mono- and binuclear Ru(II) complexes containing bipyridine ligands has been performed by electroabsorption (EA) spectroscopy. The EA spectra of Ru complexes sensitizing a TiO_2_ semiconductor were compared with the spectra of these complexes in the form of solid neat films, both of which parametrized within the Liptay theory. The extracted values of relevant parameters, relating to molecular dipoles after optical MLCT (metal-to-ligand-charge-transfer) excitation, exhibit a clearly noticeable increase for Ru complexes adsorbed on TiO_2_, but they are too small to be attributed to excitation associated with the direct transfer of an electron from the dye adsorbate to the TiO_2_ semiconductor. Due to the difficulties arising from standard analysis based on Liptay formalism, we have for the first time successfully reproduced the EA spectra of the Ru/TiO_2_ systems using the time-dependent density functional (TDDFT) calculations, incorporating into the Hamiltonian a term describing the interaction of a molecule with the local electric field it experiences from the TiO_2_ structure and the neighboring Ru complex molecules, without additional assumptions about the lineshape of the EA signal. The implications of these results are briefly discussed in the context of dye-sensitized solar cells.

**Supplementary Information:**

The online version contains supplementary material available at 10.1038/s41598-024-81957-z.

## Introduction

Ruthenium Ru(II) complexes are an important group of materials widely used as solar sensitizers and photoredox agents, in dye-sensitized solar cells (DSSCs) that efficiently convert sunlight into electricity, and in dye-sensitized photoelectrochemical water-splitting cells (DSPECs) that produce environmentally friendly hydrogen fuel^1–3^. The most successful devices of this type are equipped with photoanodes in the form of a layer of nanocrystalline semiconductor (usually TiO_2_), on the surface of which molecules of Ru dyes are adsorbed with properly designed anchoring groups, acting as a photosensitizer in the process of electron injection from the excited state of dye to the conduction band (CB) of the semiconductor. The versatility of Ru(II) complexes, in particular Ru polypyridyl complexes, is mainly due to their strong MLCT (metal-to-ligand charge transfer) excited states involved in the absorption of visible light. The MLCT states with the Ru 4d electron transferred to the π* orbitals of the ligands have been extensively studied experimentally and theoretically for five decades^4,5^. Based on transient absorption measurements, there is general agreement that photoinduced electron injection from Ru dye into TiO_2_ consists of an ultrafast < 100 fs component from the non-thermalized excited state and slower components on picosecond and longer time scales originating from the thermalized excited state, and the appropriate contribution of these two components depends on the relative energy between the excited states of the dye and the conduction band edge of the semiconductor^6^. According to the theory, the high rate of interfacial electron transfer at the photoanode surface requires strong electronic coupling between the wavefunctions of the photoexcited Ru complex and the CB states of the semiconductor. To design and develop more efficient and stable dye-sensitized devices, it is crucial to have an in-depth understanding of the photoexcitation dynamics and charge separation mechanism for the dye sensitizers attached to TiO_2_ semiconductor.

In the systems considered here, the contribution of TiO_2_ ionic states into the primary (Franck–Condon) excited state, generated immediately after absorption of the photon by the dye molecule, is of special importance and can be recognized as a large change in the permanent dipole moment upon photoexcitation. In this respect, a unique ability has electroabsorption spectroscopy (EA), in which the changes in optical absorption spectra are recorded as a result of the interaction of molecular dipoles with an applied external electric field. EA spectroscopy (Stark effect spectroscopy) is undoubtedly an effective method specifically dedicated to the detection of excited states endowed with a large permanent dipole moment that arises from charge-transfer processes^7^. So far, this method has been applied to Ru N719 dye/TiO_2_^8^, and some pure organic dyes (alizarin, catechol, anthracene, carotenoid dyes, pyrene) adsorbed on TiO_2_^9–13^, where quantitative analysis of EA spectra is based on Liptay formalism^14^. The magnitude of the change in electric dipole moment Δµ following absorption of N719/ TiO_2_ system estimated in this way is much larger than that of the solid film, suggesting that the interfacial charge transfer occurs after photoexcitation of the N719 molecule adsorbed on the TiO_2_ surface^8^. Similarly, for anthracene^11^ and pyrene^13^ molecules chemisorbed by carboxy groups on colloidal TiO_2_, in glassy ethanol at low temperature, changes in the dipole moment upon electronic excitation, Δµ, with a value of several debyes, are interpreted as indicators of partial charge transfer character of excited states, corresponding to the optical electron transfer from the adsorbate to TiO_2_. In turn, the higher value of Δµ of adsorbed alizarin was explained by the interaction of the adsorbed dye with the internal electric field generated by the charged TiO_2_ nanoparticles, rather than by the alizarin-to-TiO_2_ electron transfer process^9^.

**Fig. 1:**
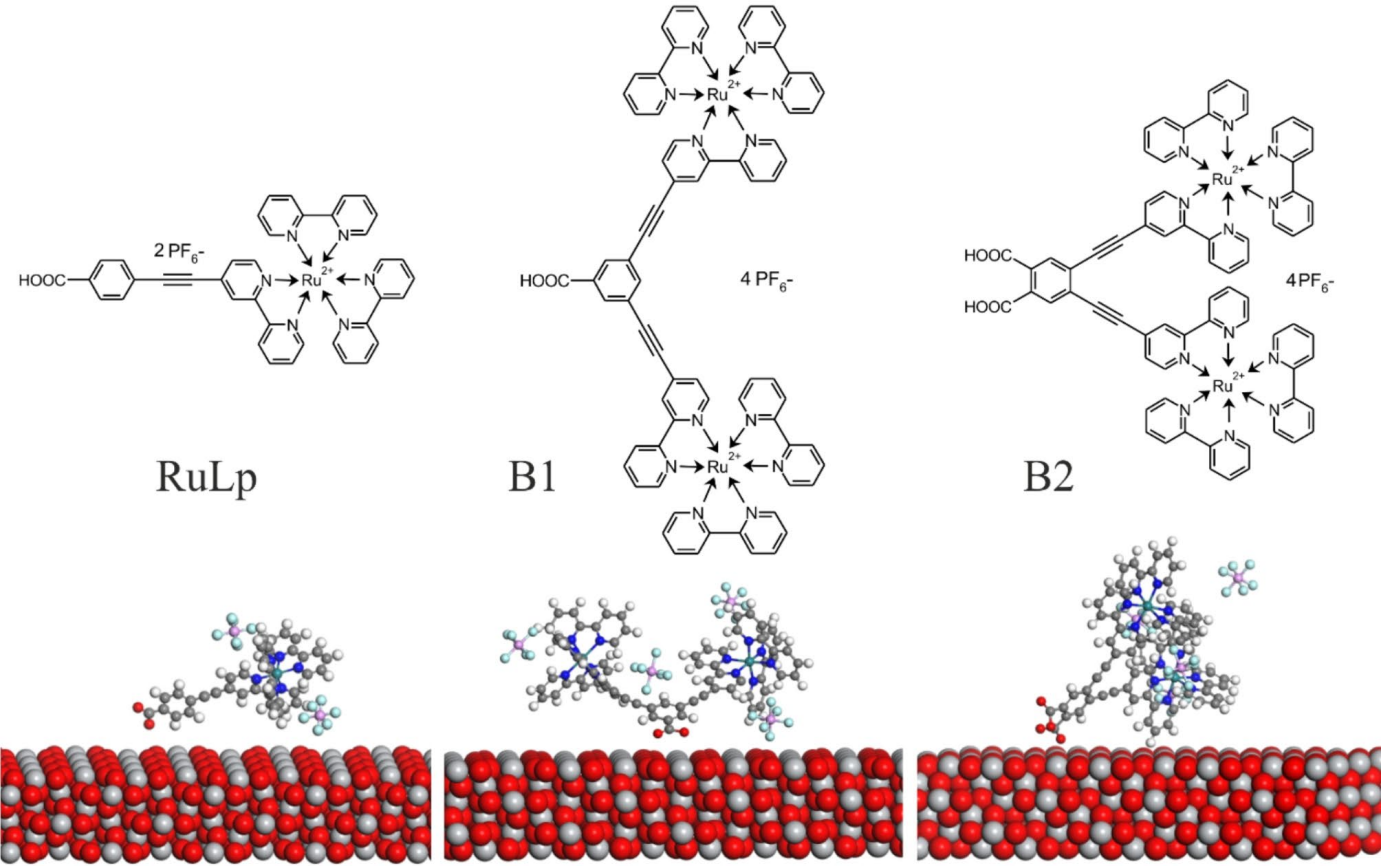
Molecular structures of the studied dyes and the position of the molecules of these dyes adsorbed on solid TiO_2_ determined on the basis of Monte Carlo simulations (see text for details).

In our previous papers^15,16^ it was demonstrated the photoelectric properties of DSSCs based on biruthenium complexes compared to the more common monoruthenium bipyridine complexes with different molecular architectures. Based on our findings, we proposed a mechanism of the electron transfer pathway underlying the electron injection process at the dye-semiconductor interface. Accordingly, we have identified certain physical factors, such as the structure of the anchoring ligand, especially the number and position of COOH anchoring groups, and the extension of the π-electrons cloud, which play an important role in the efficient operation of DSSCs. However, it remains an open question of how strongly the TiO_2_ semiconductor modifies the primary electronic states of the dye molecule involved in the act of light absorption. To solve this problem, in this work we apply EA spectroscopy and compare EA spectra measured for mono- and biruthenium bipyridine complexes in the form of monolayers of molecules of these complexes adsorbed on the surface of nanocrystalline TiO_2_ and neat films without contact with TiO_2_. The selected molecules (Fig. 1) can serve as a model for the dye-metal oxide interface in DSSCs. In addition to the typical analysis based on the Liptay formalism of the Stark effect^14^, we present for the first time the EA spectra of the Ru dye molecules attached to the TiO_2_ surface using quantum chemical calculations based on the time-dependent density functional (TDDFT) method.

## Electroabsorption

In a molecular system, under the action of an external electric field, the absorption bands generally on the energy scale broaden, shift and change in intensity. Using the standard EA spectral analysis, based on Liptay theory^14^ for well-separated and non-degenerate electronic states, the total electro-optic effect can be quantified in terms of molecular parameters. An electric field ($\:\overrightarrow{F}$) changes the absorption band of a set of molecules with a fixed orientation by the shift, Δ*E*($\:\overrightarrow{F}$), of the energy (*E*), of a single electronic transition, due to the Stark effect,

1 $$\:\varDelta\:E\left(\overrightarrow{F}\right)=\:-\overrightarrow{\varDelta\:{\upmu\:}}\cdot\:\overrightarrow{F}-\:\frac{1}{2}\overrightarrow{F}\circ\:\varDelta\:\varvec{p}\circ\:\overrightarrow{F}\:\:,$$

where $\:\overrightarrow{\varDelta\:{\upmu\:}}$ is the change in permanent dipole moment and **Δ*p*** is the change in electronic polarizability tensor after the transition from the ground state to the excited non-degenerate state. If the change in absorbance (optical density ΔD) induced by the electric field is sufficiently small, it can be developed into a Maclaurin series with respect to energy and truncated beyond the quadratic term. Assuming that the oscillator strength of the electronic transition is independent of the electric field, the measured values of Δ*D* (*E*, *F*) can be related to the averaged shift < Δ*E* > and the averaged broadening < (Δ*E*)^2^> of the absorption band. Thus, the average of Δ*D* for the isotropic ensemble of molecules is given by^17,18^

2 $$\:{\Delta\:}D=\:\frac{1}{2}\left(\:{\Delta\:}p\:\frac{\text{d}D}{\text{d}E}\:+\:{\upkappa\:}\:{\left({\Delta\:}{\upmu\:}\right)}^{2}\frac{{\text{d}}^{2}D}{\text{d}{E}^{2}}\right)\cdot\:{F}^{2}=B\left(E\right)\cdot\:{F}^{2}\:,$$

where the exact value of κ coefficient depends on the averaging procedure and the type of electronic state (non-degenerate or degenerate). Here, *B* stands for the function expressing the spectral dependence of Δ*D*. The formula (2) was derived for a beam of non-polarized light incident perpendicularly on a molecular layer equipped with sandwich electrodes and is essentially consistent with Liptay formalism^14^. In this article the κ = 1/3 was assumed to evaluate and compare Δµ in all the cases considered (see Ref^18^. for details). The independence of oscillator strength from the applied electric field strength in the derivation of formula (2) has been suggested previously for Ru complexes (see, for example^19,20^).Therefore, the changes in the average electronic polarizability (${\Delta\:}p=\overline{{\Delta\:}p}$) and permanent dipole moment (Δµ=$\:\left|\overrightarrow{{\Delta\:}{\upmu\:}}\right|$) upon photoexcitation can be determined by comparing the measured EA spectra with the spectra of the first and second-order energy derivatives of the ordinary absorption spectrum, respectively. In our laboratory, for example, this method was applied successfully to archetypical complexes applied in organic electronics, such as 8hydroxyquinoline aluminum(III) (Alq3)^21^, 2-phenylpyridine iridium(III) (Ir(ppy)_3_)^22^ and Pt(II) octaethylporphyrin (PtOEP)^23^, and more recently for mono-(RuLp) and biruthenium complexes^15,18^ in the form of neat films prepared by vacuum evaporation or spin coating.

In the context of Ru complexes studied here, a prototypical molecular structure is the trischelate [Ru(II)(bpy)_3_]^2+^ ion (hereafter referred to as RBY with 2,2’-bipyridine (bpy) ligands). In the first (lowest energy) MLCT manifold absorption band, assuming a localized model of MLCT states, for a RBY(PF_6_)_2_ complex^24^ or a RBY(Cl)_2_⋅6H_2_O adduct^25^, both dissolved in polyvinyl alcohol (PVA) matrix, two or three polar electronic states were recognized. The question is whether the initially photoexcited MLCT state is localized only on one of the bpy ligands due to certain distortion or whether it is spread over three equivalent ligands, preserving the D_3_ ground state symmetry - for a discussion of the problem and literature review see Ref^18^.

Due to the derivative character of EA spectra (see formula (2)), EA spectroscopy is a useful method for detecting spectral features of electronic excitations that are otherwise difficult to observe. In particular, when a non-zero permanent dipole is involved, an EA spectrum similar to that of the second derivative absorption spectrum makes it possible, as is often assumed, to assess the change in the permanent dipole moment after photoexcitation. Note, however, that such a quantitative assessment is valid only within the framework of the Liptay theory. In general, comparison of the EA spectrum with derivatives of the absorption spectrum is not always adequate to reliably describe the electronic transitions under study. First, the case of degenerate (by symmetry) or quasi-degenerate state requires careful treatment. Secondly, the standard Liptay formalism can also be questionable when the absorption spectrum is highly congested by electronic/vibronic states with different properties, which is the case for Ru complexes studied here. For example, at least 9 electronic states are involved in the spectral of 6000 cm^− 1^ range of the first MLCT absorption band of the RBY complex^5^, not counting their various vibrational replicas and triplet states. The coexistence of multiple excited states hidden in the broad and poorly resolved spectrum means that the overall EA response of the molecular system is a function of too many parameters to be unambiguously estimated. Besides, in the case of significant mixing of closely lying excited states by an applied electric field, the Liptay formalism itself is problematic because it relies on the calculation of small quantum-chemical perturbations. In such a case, the EA spectra should be rather determined directly by definition as the difference between the absorption spectra computed in a nonzero and zero electric field without arbitrary assumptions about the shape of the spectral features, as proposed originally for polyacene films^26^ and recently by our group for ruthenium complexes^15,18^. Therefore, in the present work, in addition to the discrete parametrization of EA spectra carried out under the standard decomposition procedure using Liptay formalism, the experimental EA spectra have been compared with spectra computed by the quantum chemical method. In this approach, the system’s Hamiltonian contains the term describing the interaction of the molecule with the local electric field it experiences, without additional arbitrary assumptions about the molecular parameters of the individual excited states and electric field correction factor (*f*). The local electric field is computed rigorously by directly considering the molecular environment, without resorting to a continuum medium or mean-field approximations, taking into account the influence of point charges located on the surrounding atoms of neighboring Ru molecules and the TiO_2_ structure.

## Results and discussion

**Fig. 2:**
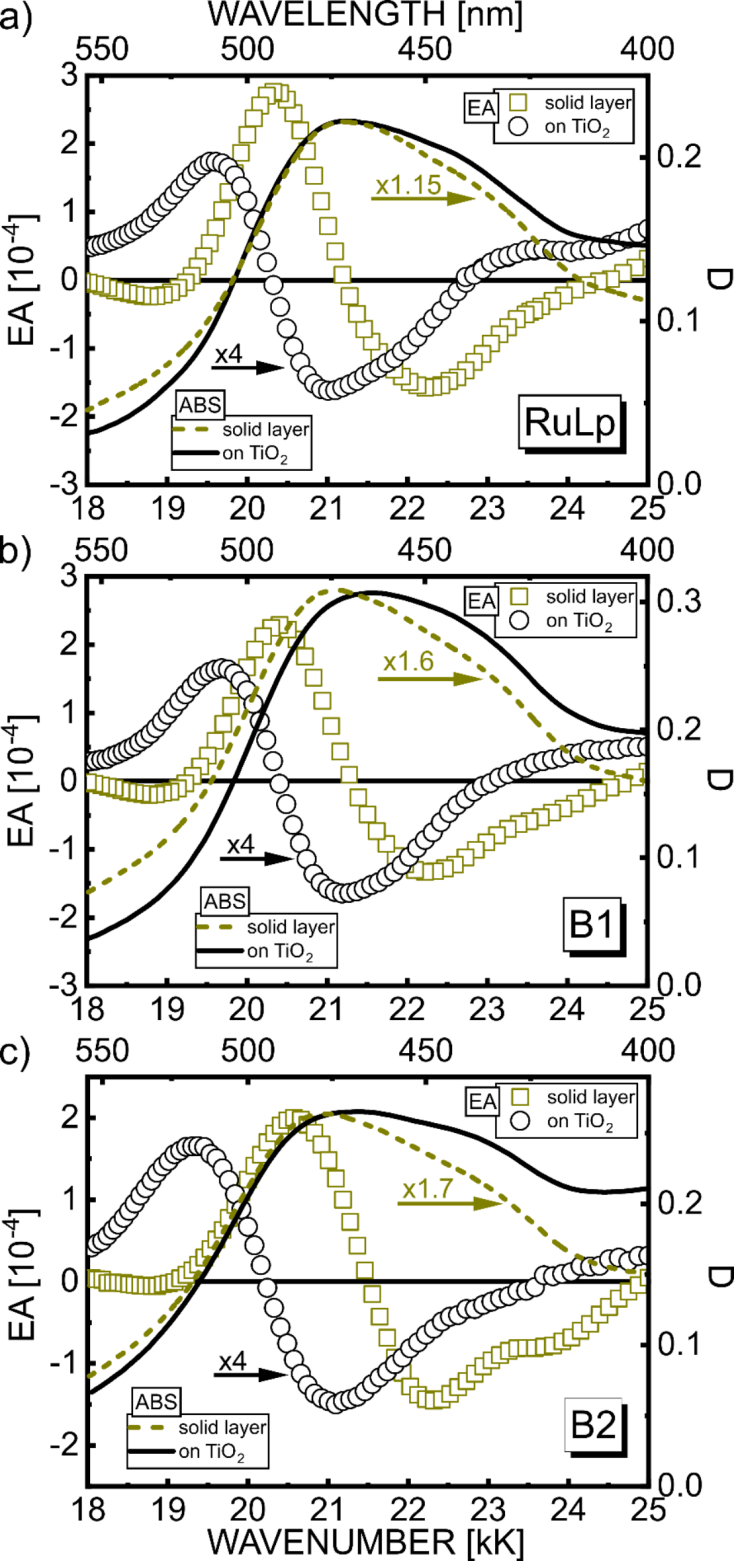
Experimental EA spectra of Ru complexes (RuLp, B1, B2) adsorbed on TiO_2_ (circles, $\:{F}_{\text{r}\text{m}\text{s}}=2\cdot\:{10}^{5}\text{V}/\text{c}\text{m}$) in comparison with those spectra measured on solid neat layers (squares, $\:{F}_{\text{r}\text{m}\text{s}}=6\cdot\:{10}^{5}\text{V}/\text{c}\text{m}$). The absorption spectra (optical density D) for these systems are displayed with solid and dashed lines, respectively. Wavenumbers are expressed in kilokaysers, 1kK=1000 cm^−1^.

Absorption spectra of three compounds RuLp(PF_6_)_2_, B1(PF_6_)_4_ and B2(PF_6_)_4_, in the form of solid neat films are very similar in the spectral range 18 kK – 25 kK (dashed lines in Fig. 2, see also Fig. 1S in Supplementary materials). As confirmed by our previous TDDFT calculations^18^, electronic transitions in this region can be assigned to the absorption of MLCT (1) origin (hereafter denoted MLCT). The introduction of a functional anchoring group in RuLp(PF_6_)_2_ disrupts the D3 symmetry properties of the electronic RBY system, and consequently, the number of excited states carrying noticeable absorption intensity increases in RuLp(PF_6_)_2_ because the degeneracy of E-type excited states is lifted and some low-intensity A-type states gain oscillator strengths by interacting with other states. Although the excited states’ level pattern is more complicated in biruthenium complexes, the main spectral features observed in monoruthenium are still preserved in biruthenium complexes. This is due to rather weak electronic coupling between the two constituent RBY units of the biruthenium molecule, which makes the contribution of heterogeneous MLCT transitions associated with electron transfer from the Ru-centered molecular orbital (MO) of one RBY unit to the bpy ligands of the other RBY unit rather weak. The simple energy level scheme can be recognized for the main MLCT state at about 22 kK, well distant from others in RBY – this state is still degenerate in RBY(PF_6_)_2_ but split into two and four components in RuLp(PF_6_)_2_ and B1(PF_6_)_4_, respectively.

Basically, the absorption spectra of Ru complexes adsorbed on the nanocrystalline TiO_2_ layer (solid lines in Fig. 2) show only minor differences compared to those spectra of neat solid films. Greater differences can be seen between the EA spectra of Ru complexes (Fig. 2) on TiO_2_ (circles) and without TiO_2_ (squares). Although both have similar shape, the main negative EA lobe for systems with TiO_2_ is shifted about 1 kK towards lower energies compared to systems without TiO_2_, which suggests that the involved MLCT states are somehow modified by the interaction of the Ru molecule with TiO_2_. However, this interaction is rather modest, and the magnitudes of EA signals are of a similar order; more importantly, the electric field applied to the Ru complex/TiO_2_-samples does not reveal any new interesting features that stand out from the broad absorption band. Our comprehensive analysis based on both Liptay formalism and quantum chemical calculations performed in this work fully supports this statement.

**Fig. 3:**
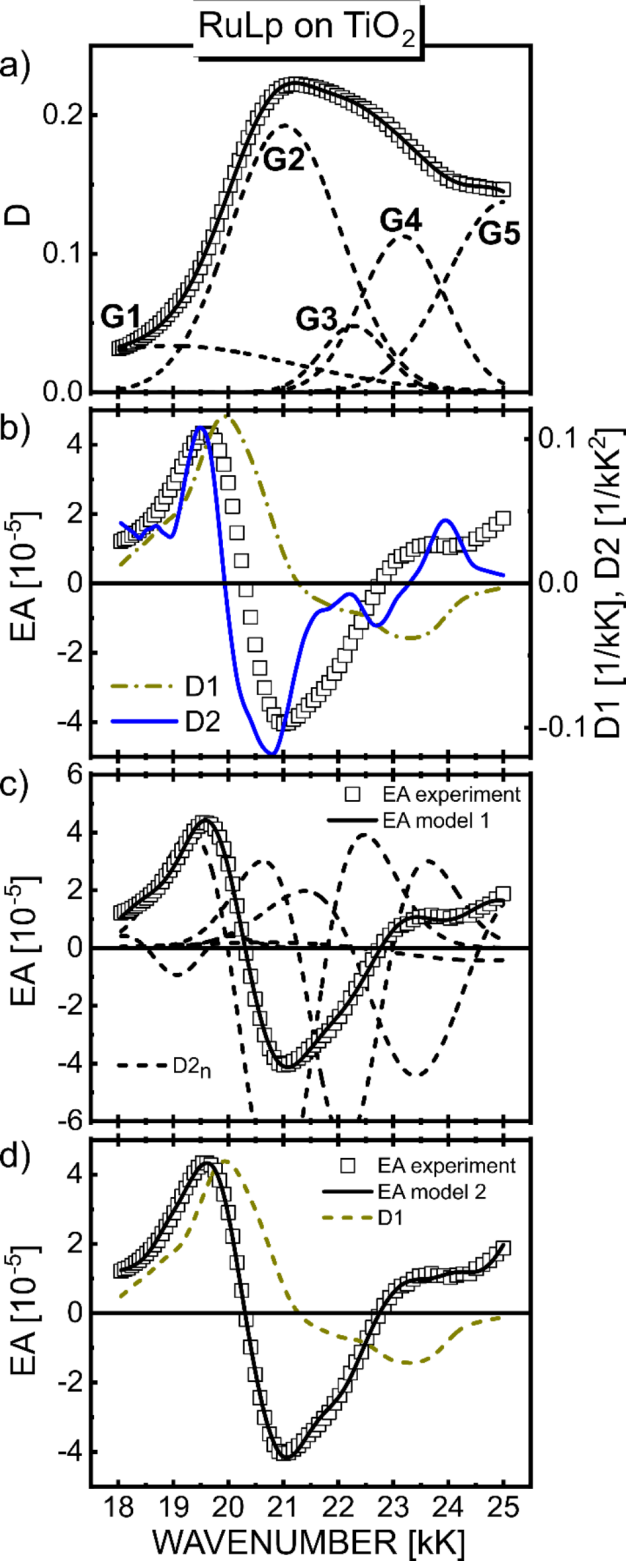
The experimental EA spectrum of RuLp/TiO_2_ system (squares, $\:{F}_{\text{r}\text{m}\text{s}}=2\cdot\:{10}^{5}\text{V}/\text{c}\text{m}$) compared with the global absorption derivatives D1 and D2 (**b**), and theoretical curves (solid lines) calculated using model 1 (**c**) and model 2 (**d**). In part (**a**) the absorption spectrum (squares) was decomposed to superposition (solid line) of the Gaussian profiles (dashed lines). In parts, (c) and (d), the dashed lines indicate the contributions to the EA signal of the second absorption derivatives (D2_n_ = d^2^D_n_/dE^2^) of the nth individual Gaussian and the first global absorption derivative (D1), respectively.

**Fig. 4:**
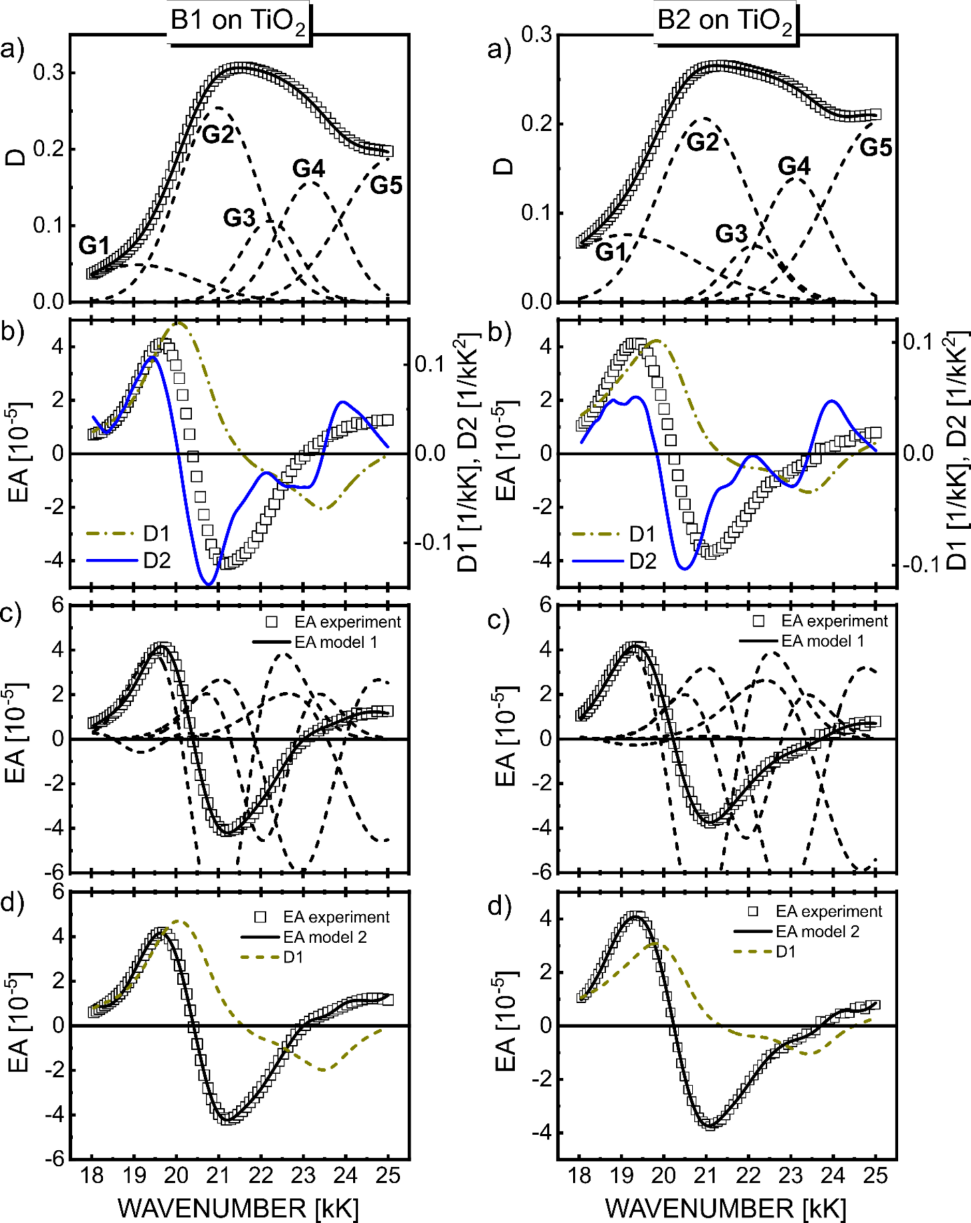
A set of experimental and theoretical results based on Liptay formalism, analogous to Fig. 3, for biruthenium complexes B1 (left side) and B2 (right side). $\:{F}_{\text{r}\text{m}\text{s}}=2\cdot\:{10}^{5}\text{V}/\text{c}\text{m}.$

Direct comparison of the EA spectrum with the absorption energy derivatives based on formula (2) provides information about changes in dipole moment (Δµ) and polarizability (Δ*p*). All RBY-based mono- and biruthenium complexes exhibit EA spectra with a shape mimicking the absorption second derivative with a negative lobe minimum at approximately 21 kK (Figs. 3b and 4b). This common feature of EA was attributed to a highly delocalized excited state of MLCT origin, somewhat resistant to modifications of the peripheral parts of bpy ligands^18^.

Then a full standard analysis based on Liptay theory was performed, decomposing ($\:D=\sum_{n}{D}_{n}$) the absorption spectra into five Gaussian bands, and then parametrizing the EA spectra using Eq. (6) in model 1 which neglects the polarizability contributions, or Eq. (7) in model 2 which assumes the same polarizability coefficients for all electronic transitions involved. The latter assumption is made quite arbitrarily in order to reduce the number of fitting parameters, but it seems reasonable when all excited states in the considered, not very broad, spectral region belong to the same MLCT manifold. The best-fit curves obtained in model 1 are shown in Fig. 3c for RuLp/TiO_2_ system (solid lines), and in Fig. 4c for B1, B2/TiO_2_ system, where the individual contributions from G1-G5 bands are also displayed (dashed lines). Figures 3d and 4d, in turn, compare the best theoretical curves based on model 2 (solid lines) with the contributions to the EA signal from the first derivative term in Eq. (7) shown by the dashed lines. All results of EA parametrization are listed in Table 1S for all three complexes, and the extracted values of changes in the permanent dipole moment and electronic polarizability are collected in Tables 1 and 2, respectively, including the local field coefficient *f*, i.e., they actually represent *f*⋅Δµ and *f* ^2^⋅Δ*p*. The values in the tables are given with two significant digits, i.e., with a relative error of 10%. Since it is difficult to unambiguously evaluate the heights *D*_0*n*_ for all Gaussians buried in the total MLCT absorption envelope exhibiting only slight features, the Δµ_*n*_ dipole moments were therefore calculated in two ways: first based on the amplitudes *D*_0*n*_ taken from the Gaussian bands shown in parts (a) of Figs. 3 and 4, and then assuming that the amplitudes are equal to the total absorbance *D* at the wavenumber position of *n*th Gaussian maximum. The Δµ_*n*_ values obtained by the latter method are presented in Table 1 in parentheses for both models. The broad G1 band in the lower energy tail of the absorption spectra can generally be ascribed to the direct singlet-triplet MLCT absorption^4,5^. Since the contribution of this band to the EA fitting curve in our work is relatively small in all three complexes, and this low absorption region may also be undesirably distorted by light scattering - this spectral region will not be analyzed further.

**Table 1:** Dipole moment changes, *f*·Δµ (in debyes), in the spectral range of G2-G4 bands. The uncertainty of parameter is around 10%.

Ru complex	Band	Solid film				Adsorbed on TiO_2_			
		Model 1		Model 2		Model 1		Model 2	
		*f*·Δµ	(*f*·Δµ)_av_	*f*·Δµ	(*f*·Δµ)_av_	*f*·Δµ	(*f*·Δµ)_av_	*f*·Δµ	(*f*·Δµ)_av_
RuLp	2	3.8 (3.6)		3.2 (3.1)		10 (12)		12 (15)	
	3	16 (7.9)	9.3 (6.1)	12 (6.1)	7.3 (4.9)	17 (10)	13 (11)	10 (6.2)	11 (11)
	4	8.0 (6.7)		6.8 (5.5)		12 (12)		11 (12)	
B1	2	3.4 (3.2)		2.9 (2.8)		8.5 (10)		13 (15)	
	3	11 (5.1)	9.5 (6.4)	12 (5.5)	7.6 (5.0)	8.3 (6.4)	9.3 (9.1)	7.7 (5.9)	11 (11)
	4	14 (11)		8.0 (6.6)		11 (11)		11 (11)	
B2	2	2.8 (3.2)		2.0 (2.4)		9.8 (11)		12 (13)	
	3	9.8 (7.0)	9.2 (8.7)	11 (7.8)	7.7 (7.1)	11 (7.1)	11 (10)	10 (6.5)	11 (11)
	4	15 (16)		10 (11)		12 (12)		12 (12)	

We can see that although the average values of dipole moments for G2-G4 bands in the MLCT absorption range, (*f*⋅Δµ)_av_, for the Ru dyes adsorbed on TiO_2_, exhibit a rather modest increase compared to solid films, the excited-minus-ground state dipole moments, Δµ_*2*_, for the leading absorption band (G2) increases remarkably about 3–4 times after adsorption of Ru dye molecules on TiO_2_. A very similar effect was observed for N719 Ru dye^8^ and pure organic molecules such as aromatic hydrocarbons (anthracene^11^, pyrene^13^). This means that despite the observed similarity of the absorption spectra, it is worth conducting a quantitative analysis of the EA spectra, as it indicates a clear effect of the TiO_2_ semiconductor on the excited states of the dye molecules involving the redistribution of charge in the excited state. However, even the maximal value of dipole moment changes upon photoexcitation found in Table 1, namely, 13 (15) debyes for the G2 band in the B1 biruthenium complex in model 2, is definitely too small to attribute this excitation to direct electron transfer from the dye molecule to TiO_2_ semiconductor. Full electron transfer over a distance of 740 pm, i.e., the shortest distance between the TiO_2_ surface and the Ru^2+^ ion estimated in the B1/TiO_2_ system^27^, yields a charge-transfer dipole moment of about 36 debyes – such a high permanent dipole moment would be easily detectable in an EA spectrum due to the large EA signal with sharp specific spectral lobes in the shape of the second derivative of the absorption spectrum, which is not the case here. It should also be noted that the above estimate of dipole moments *Δµ* for Ru dyes should be treated with caution due to the somewhat complicated pattern of interaction with the electric field of several excited states involved in the EA response, which was identified using quantum chemical calculations (see below).

**Table 2:** Polarizability changes, *f*^2^·∆p (in Å^3^), in the spectral range of G2-G4 bands. The uncertainty of ∆p parameter is around 10%.

Ru complex	Solid film	On TiO_2_
RuLp	70	140
B1	90	170
B2	90	130

The reliability of the values of Δ*p* extracted from EA spectra based on model 2 (Table 2) is even less certain. This is especially the case for polar excited states when EA spectra are mainly governed by the second derivative terms in Eq. (7). The Δ*p* values for Ru dye/TiO_2_ systems about 2 times higher than in solid films were obtained, and in this respect the electronic behavior of mono- and biruthenium complexes appears similar. In comparison, Ohta and coworkers^8^ reported about a tenfold increase in this magnitude for the Ru N719 dye/TiO_2_ system compared to solid films, but the values extracted from their EA spectra of solid neat films were only 9 Å^3^, so despite the same two bpy ligands of Ru N719 complex, about 10 times less than in the Ru complexes, containing three or six bpy ligands, considered in the present paper. Of course, the higher Δ*p* values for dye-sensitizer/semiconductor systems, compared to systems without TiO_2_, can be easily justified in terms of the more extended electronic clouds of MO interacting with the CB states of the semiconductor. In this context, it is worth to recall the paper^11^ on anthracene-9-carboxylic acid (ACA), where anthracene molecules were anchored to TiO_2_ by the carboxy groups, and the number of states participating in the EA signal is significantly smaller than in RBY-based complexes. In this case, given the rather small and strongly variable first derivative terms, as the authors of the paper^11^ found, the change in electronic polarizability Δ*p* for the adsorbed forms of ACA could not be estimated at all. As a side note, it is worth mentioning that in the discussed work, based on EA spectra of a free carboxyanthracene molecule (ACA) dissolved in glassy ethanol, a value of Δ*p* ≅ 160 Å^3^ was extracted for the change in polarizability after excitation of the first lowest energy singlet state, where this value is ascribed to the presence of the large polarizability component down the long molecular axis and perpendicular to the transition moment of the ACA molecule. In such a case of a molecule with polarizability in one direction much larger than the perpendicular polarizability components, the tensor, **Δ*p*** reduces to a scalar (uniaxial) polarizability Δ*p*^9,10^. For comparison, over 4 times lower value of the electronic polarizability estimated from EA measurements performed for unsubstituted (and therefore non-polar) anthracene molecules dissolved in the polymer matrix^28^, confirming again this way the difficulty of EA analysis and proper assessment of the tensor (directional) properties of **Δ*p*** (for details see also Ref^7^).

**Fig. 5:**
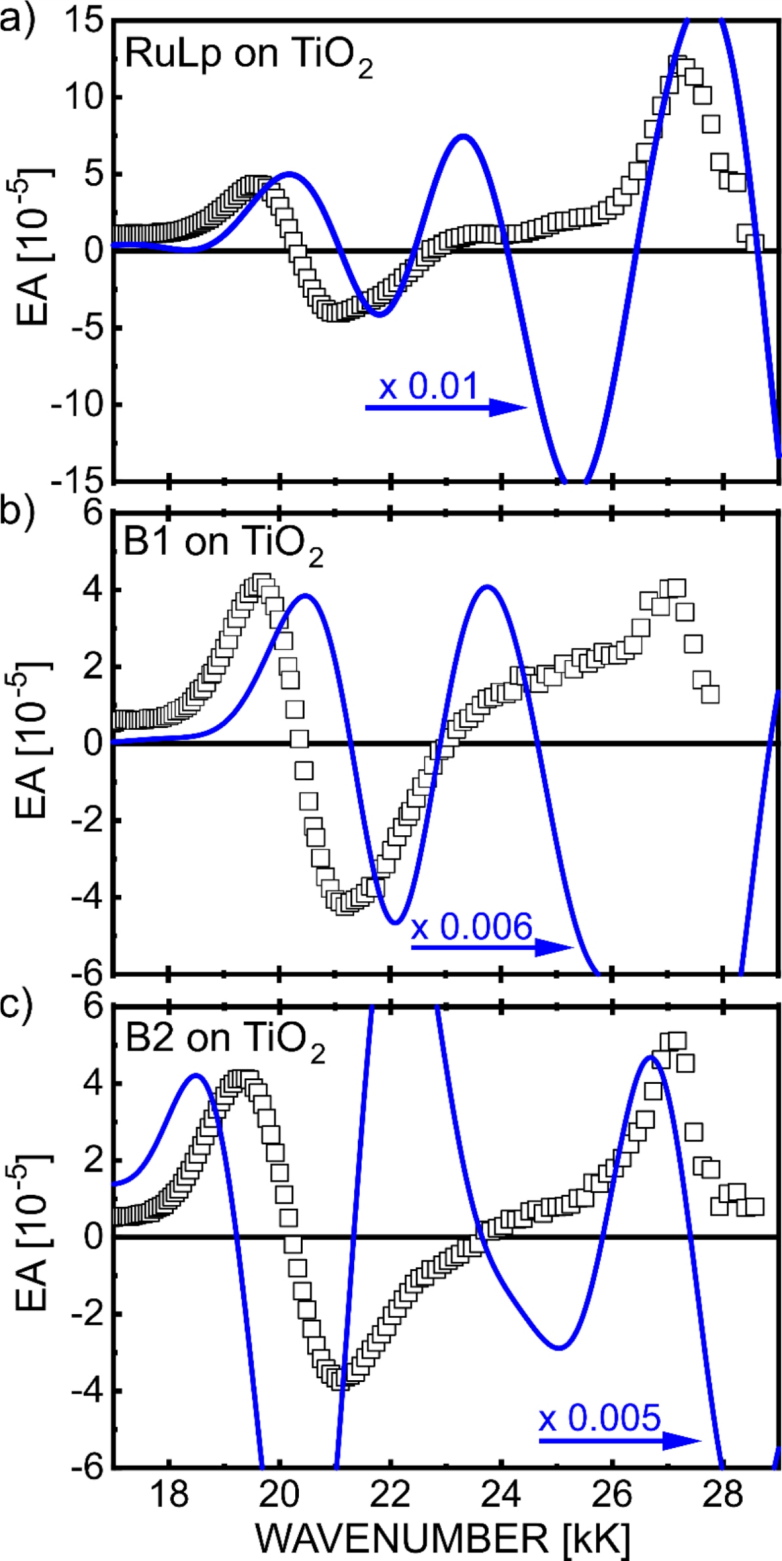
The EA spectra calculated using the TDDFT method (solid lines, F = 5.14·10^6^ V/cm) compared with the experimental EA data (squares, F_rms_ = 2.2·10^5^ V/cm) for systems containing RuLp (**a**), B1 (**b**), and B2 (**c**). The Gaussian bandwidths *w* = 2.0 kK.

As mentioned earlier, the standard decomposition of EA spectra based on Liptay theory into a multicomponent linear combination of derivatives of individual absorption profiles is difficult to reliably perform in spectral ranges with many near-distant excited states on the energy scale. Therefore, in Fig. 5, experimental EA spectra of Ru complex/TiO_2_ systems for monoruthenium RuLp and biruthenium (B1, B2) complexes (squares) are compared with EA curves (solid lines) computed by the TDDFT method. As described earlier discussing Fig. 2, the absorption spectra of Ru complexes adsorbed on TiO_2_ show only minor differences in signal magnitude and spectral shape compared to those spectra of neat solid films. Consequently, weak electronic coupling was assumed between the dye and the semiconductor, treating the local electric field as a perturbation. Thus, a term describing the interaction of the molecule with the electric field from the environment consisting of the TiO_2_ surface and neighboring molecules of the Ru complex was included in the system’s Hamiltonian. As can be seen, the spectral dependence of the EA signal in the 18–24 kK range, which includes the main low-energy absorption of MLCT character, is roughly reproduced by TDDFT calculations (for details of calculations, see Sect. on TDDFT method). As can be seen in Fig. 5, the values of experimental EA signals measured at an electric field strength of 2.2·10^5^ V/cm are more than two orders of magnitude lower than the theoretical EA signals calculated for 5.14·10^6^ V/cm. The electroabsorption mechanism following the Stark effect (see formula (2)) yields an experimentally confirmed linear dependence of EA signals on the square of the applied electric field. A further discussion of the validity of such an approximation in Ru complexes can be found in our earlier work^18^. If the calculated EA signals are scaled to the value of the experimental electric field of 2.2·10^5^ V/cm, taking into account the quadratic dependence of EA signals on the field, they are actually 5.5, 3.3 and 2.5 times smaller, respectively, in RuLp, B1 and B2. This is indeed a very satisfying result if we consider the validity of the assumptions made in the calculations, such as the linear dependence of absorbance on concentration found in the Lambert-Beer formula (8), the rather simplified local electric field model concerning partial charges located on neighboring atoms, the dependence of EA signals from the second power of the applied electric field, to name a few more important ones. The assumed bandwidth *w* = 2 kK = 2000 cm^− 1^ is consistent with the average width of G2-G4 Gaussians extracted from experimental global absorption spectra (Figs. 3a and 4a). This value seems reasonable given the dominant vibrational progression with an energy interval of 1300–1500 cm^− 1^, equivalent to aromatic ring stretching modes and other lower frequency modes and their replicas active in the infrared and Raman spectra of the RBY cation^29^.

## Conclusions

An explanation of the charge-transfer effects occurring at the Ru dye/TiO_2_ interface lies at the heart of DSSC operation. As discussed in Introduction section, it is generally accepted that photoinduced electron transfer from the Ru dye to the TiO_2_ semiconductor is a two-step process, in which the final electron injection occurs as a result of the ultrafast (on the femtosecond scale) dissociation of the primary excited state of the dye molecule. On the other hand, there are some results of quantum chemical calculations for the Ru dye/TiO_2_ systems suggesting that some excited states’ orbitals, in particular, LUMOs (Lowest Unoccupied Molecular Orbital), are located on the TiO_2_ part of such hybrids, forcing direct electron transfer^30,31^. The latter case, however, is difficult to substantiate reliably. First, the theoretical oscillator strength for such lowest-energy CT transitions is too weak to detect them spectroscopically^31^, at least under broadband spectroscopy conditions. Second, current limitations of computer programs only allow the implementation of TiO_2_ clusters which are much smaller compared to actual TiO_2_ nanoparticles – so the predicted theoretical results may deviate from reality. However, the properties of the primary (Franck-Condon) excited states, generated immediately after photon absorption by a dye molecule attached to the surface of a TiO_2_ nanoparticle, can be directly identified and characterized by EA spectroscopy, which was the goal of this work.

Accordingly, the EA spectra of Ru complexes on a TiO_2_ semiconductor were parametrized using standard analysis based on Liptay theory. Although the extracted values of the permanent dipole moments related to optical MLCT excitations show a clearly noticeable increase for Ru complexes adsorbed on TiO_2_, they are far too small to be attributed to excitation associated with direct electron transfer from the dye molecule to the TiO_2_ semiconductor. As an alternative, the TDDFT method was used, which allows us to consider the collective response of all excited states involved in the spectrum without additional assumptions on the EA signal lineshape from individual electron transitions. In this approach, which is free of standard field-correction factors (*f*) for a molecule embedded in a vacuum cavity in a continuous medium, the influence of the environment in the weak electron-coupling regime was taken into account by considering the explicit interaction of the dye molecule with the local discrete electric field from neighboring molecules and the TiO_2_ structure. The spectral dependence and even the magnitude of the EA signals were reproduced quite well, suggesting that our interpretation of the EA spectra, despite the many approximations made - to those previously mentioned it is worth adding the neglect of triplet excited states - is substantively correct and can be applied and tested in the future on other dye sensitizers.

## Methods

### Sample preparation

The syntheses of ruthenium complexes RuLp, B1 and B2 were described in articles^15,16^. Optical absorption was measured on quartz/Ru dye or glass/FTO/TiO_2_/Ru dye samples. For electroabsorption (EA) measurements, a sandwich structure, glass/FTO/TiO_2_/Ru dye/PMMA/Al, with FTO (fluorine doped tin oxide) and Al as electrodes was used. The solid neat films of Ru dyes were formed by spinning a solution of Ru material in acetonitrile at 1000 rpm, obtaining smooth films with a thickness of 40–60 nm. A colloidal paste containing TiO_2_ nanoparticles approximately 20 nm in diameter (DSL 18NR-T), after being dissolved in ethanol (99,8%) was deposited on an FTO-covered glass substrate by spinning at 500 rpm. The resulting 2.3 µm-thick TiO_2_ layer was then gradually heated to 500 °C for one hour and then cooled to about 80 °C. This procedure, essentially similar to that used in Refs^32,33^, yields a rather smooth anatase TiO_2_ layer. In the next step, the layer was dipped into a dye solution (≈ 0,1 mM in anhydrous ethanol) at room temperature for 16 hours to adsorb the dye. At this stage of sample preparation, a fairly thick dye layer is formed, so dye molecules not bound to TiO_2_ were removed by bathing the sample in acetonitrile and acetone, leaving only those adsorbed on TiO_2_ nanoparticles, forming a final dye monolayer after evaporation of the solvents. To mitigate any remaining roughness and prevent electrical breakdown, a 0.5 μm thick PMMA layer was deposited by dynamic spin coating at 2500 rpm. A semi-transparent aluminum (Al) electrode was deposited on Ru dye neat film or on the PMMA layer, using a vacuum vapor deposition technique. Layer thicknesses were determined using a Tencor Alpha Step 500 profiler.

### Electroabsorption measurements

The absorption spectra were recorded with a Perkin-Elmer Lambda 10 spectrophotometer. In the case of electroabsorption, signals were detected using a typical phase-sensitive technique. The light beam from the halogen lamp (Osram 64655 HLX), dispersed by a grating monochromator (Acton SpectraPro SP-2150), passed perpendicularly through the sandwich sample and parallel to the external electric field applied to it, and then detected by the Si photodiode (Hamamatsu S1337-66BR). The photodiode current was increased by preamplifier (EG&G Parc, model 181). The DC component of the electrical signal was recorded by an electrometer (Keithley, model 2000), and the AC component of it was fed to a lock-in amplifier (PAR, model 5210). The modulation frequency of the sinusoidal voltage was about 1230 Hz. The EA signals can then be determined from the formula:

3 $$\:EA\equiv\:\frac{{I}_{2\omega\:}}{{I}_{0\omega\:}}$$,

where $\:{I}_{2\omega\:}$ is the rms value of the (2ω)-Fourier component measured with a lock-in amplifier, and $\:{I}_{0\omega\:}$ is the value of the (0ω)-Fourier component of the light passing through the sample. All measurements were fully automated using the LabVIEW environment.

The experimentally recorded EA signals, defined by formula (3), are related to the signals ΔD in formula (4), by the following relationship:

4 $$\:EA=\frac{\text{l}\text{n}\left(10\right)}{2\:\:\sqrt{2}}\:{{F}_{e}}^{2}\cdot\:B\left(E\right)\:=\:\:\frac{\text{ln}\left(10\right)}{2\:\:\sqrt{2}}\:\varDelta\:D\:.$$

In formula (4), *F*_e_ = *f*⋅*U*/*d*, where *f* is the local field correction factor, and *U* is the external voltage amplitude applied to the sample of thickness *d*. In the *Lorentz approximation*, assuming the molecule is placed in a spherical vacuum cavity in a dielectric continuous medium, *f* = (ε + 2)/3, which for a typical relative permittivity, ε = 3–4, gives a value of *f* = 1.7-2.0. In turn, the *applied field approximation* usually takes *f* = 1. The method for determining the local electric field in TDDFT calculations is described below in the TDDFT section.

Numerical calculations based on Liptay formalism.

The first D1 (d*D*/d*E*) and second D2 (d^2^*D*/d*E*^2^) derivatives of the experimental absorption spectra were calculated using the Savitzky-Golay method (Origin 2021 program) and next smoothed by a local polynomial regression using the Savitzky-Golay filter. The absorption spectra were decomposed ($\:D=\sum_{n}{D}_{n}$) into several Gaussian bands,

5 $$\:{D}_{n}={D}_{0n}\:\text{e}\text{x}\text{p}\left[-4\:\text{l}\text{n}2\:{\left(E-{E}_{n}\right)}^{2\:}/\:{{w}_{n}}^{2}\:\right]\:,$$

where *w*_*n*_ = FWHM (full width at half maximum), *E*_*n*_ is the central energy position, and *D*_0*n*_ – the maximum intensity (height) of the *n*th Gaussian.

Based on the Liptay formalism, the EA spectrum can be decomposed into a linear combination of derivatives of Gaussian bands. Therefore, the EA experimental spectra were fit with theoretical curves calculated according to Eq. (6) for model 1, or Eq. (7) for model 2:

6 $$\:EA\:=\:\sum_{n}{b}_{n}\:\frac{{d}^{2}{D}_{n}}{\text{d}{E}^{2}}\:,$$

7 $$\:EA\:=\:a\:\frac{\text{d}D}{\text{d}E}\:+\:\sum_{n}{b}_{n}\:\frac{{\text{d}}^{2}{D}_{n}}{\text{d}{E}^{2}}\:.$$

In model 1, only the second derivative terms with the permanent dipole moments (Δµ_*n*_) coefficients, were considered. In model 2, the first derivative (d*D*/d*E*) of the total (experimental) absorption spectrum (*D*) was added with a single scaling factor (*a*), assuming the same change in electronic polarizability (Δ*p*) for all participating transitions. The fitting procedure was carried out using a nonlinear leastsquares method based on the Levenberg-Marquardt algorithm^34^, which determines the bestfit parameters by iteratively minimizing the merit function χ^2^ using a gradient method. The fitting parameters were coefficients, *a* and *b*_*n*_, energy positions (*E*_*n*_) and bandwidths (*w*_*n*_) of Gaussian bands. Based on the best-fit values of *a* and *b*_*n*_, taking the intensities of individual Gaussians from the ordinary absorption spectrum, the polarizability (Δ*p*) and dipole moment changes (Δµ_*n*_) were evaluated. To facilitate comparison with commonly available literature data, the Δµ values are expressed in debyes (1D = 3.34·10^–30^ C·m), and the Δ*p* values are expressed in volume units (1Å^3^ = 1.11·10^–40^ F·m^2^), where conversion factors to SI units are given in parentheses.

For convenience, the abscissa of absorption and EA spectra is expressed as the wavenumber in units of kilokaysers (1 kK = 10^3^ cm^−1^).

### Monte Carlo simulations

To find the most stable adsorption sites of the RuLp, B1 and B2 molecules on the anatase TiO_2_ (111) surface, the Monte Carlo (MC) simulations were performed using the Materials Studio program. First, the TiO_2_ (111) surface was constructed and its energy was optimized using the Forcite simulation module implementing the Universal force field^35^. The convergence criterion was chosen as 10^− 4^ kcal/mol for the energy convergence and 10^− 3^ kcal/(mol⋅Å) for the forces. In consequence, the orthorhombic unit cell with its edges equal to 37.94 Å × 21.36 Å × 66.68 Å, including the TiO_2_ (111) surface, was built. Three RuLp, or two B1, or two B2 molecules were located in the simulated unit cell to investigate their location on the TiO_2_ surface (see Fig. 1). In this case, the simulated annealing of the adsorption locator option implemented in Materials Studio was used. The temperature changed in five cycles from 10^2^ to 10^3^ K. The geometry of the system in each cycle was optimized to find the most stable structure of the created hybrid system. All computational parameters used were the same as those applied to perform geometry optimization of the TiO_2_ surface. The structures with the lowest total energy were taken to calculate the environmental influence on the electron properties of the RuLp, B1 and B2 molecules.

### TDDFT quantum chemical calculations

The geometries of the RuLp, B1, B2 and B3 molecules were optimized by looking for the minimum of their total energy by the density functional theory (DFT) method applying B3LYP functional (DFT/B3LYP)^36,37^. The 2^nd^ -order scalar relativistic effect was included within the Douglas–Kroll–Hess (DKH2) formalism^38–40^. The Jorge-TZP-DKH basis set was used for all the atoms according to our previous work^18^. The molecules were optimized in free space with stabilizers as RuLp(PF_6_)_2_ and BX(PF_6_)_4_ structures^31^.

The environmental effect changing the optical properties of RuLp, B1, or B2 molecules, including the TiO_2_ structure as well as the neighboring Ru complex molecules, was taken into consideration by applying the discrete local field method via hierarchical calculations. In this approach, molecular properties are related to their native parameters by local field factors, which describe the effect of the point charges located at surrounding atoms on a molecular site. In discrete local field theory, the local fields are computed by considering the molecular environment rigorously, without resorting to a continuum or mean-field approximations^41^. The simplest scheme used corresponds to the point-dipole approximation, where the electrical response of each molecule to the local fields induced by the externally applied fields is expressed by induced point dipoles situated in the respective molecule center of mass (COM). Because of the intramolecular flexibilities of each molecule in the MC simulations, its COM was determined anew at each considered snapshot of the trajectories.

The local electric field was calculated over trajectories, calculating the electrostatic interaction between the center of mass of the adsorbed molecule and each atom located in the calculated unit cell. Then, calculations of the local field were extended to the next coordination sphere of neighbors. The calculations of the local field stopped when between the two following coordination spheres the local field did not change more than 10^− 5^ GV/µm. The obtained local field F(x, y,z) was included in the Hamiltonian to calculate the optical properties of the RuLp, B1 and B2 molecules affected by the environment.

The vertical transition energies and oscillator strengths for the first 100 singlet excited states for the RuLp molecule and 60 singlet excited states for B1 and B2 molecules were calculated by the time-dependent DFT (TDDFT) method at the B3LYP/DKH2 level with the Jorge-TZP-DKH basis set for the molecules optimized in their ground states. The calculations were performed with an applied local electric field only for the optimized geometry, and it was assumed that the properties of molecules were the same for each geometric structure adopted during the simulations.

Due to the dominant role of singlet excited states in absorption and electroabsorption (EA), only singlet–singlet transitions were considered. The transition energies were calculated using the iterative Davidson method^42^ with an accuracy of 10^− 12^ Hartree. The theoretical EA spectra based on TDDFT were calculated by subtracting the absorption spectra without an external electric field from these spectra in the presence of an external electric field. To attain reliable EA signals that are greater than the numerical noise, TDDFT calculations were performed at an electric field strength of 0.001 au = 5.14·10^6^ V/cm, i.e., one order of magnitude higher than the applied experimental electric field. Absorption spectra for electric fields oriented along the principal directions (− x, x, −y, y, −z, z) of the molecule were averaged for the isotropic distribution of molecular orientations according to the random adsorption of molecules on TiO_2_ nanoparticles. The absorption spectra were composed ($\:D=\sum_{n}{D}_{n}$) from Gaussian profiles, *D*_*n*_, assigned to the *n*th transition, with *E*_*n*_ -the TDDFT calculated energy position, and the maximum intensity *D*_*0n*_ calculated from the TDDFT oscillator strength *f*_*n*_, assuming the validity of the Lambert-Beer law^5,18^:

8 $$\:{D}_{0n}=c\:l\:\frac{{f}_{n}}{4.6\cdot\:{10}^{-9}{\:w}_{n}}\:,$$

where *c* is the molar concentration (in mol/dm^3^), *l* is the thickness (in cm) of the monolayer of Ru dye adsorbed on spin-coated TiO_2_ nanoparticles, and *w*_*n*_ is the bandwidth (in cm^− 1^). The *l* = 22 nm was inserted into formula (8), which is equal to the diameter of TiO_2_ nanoparticles. Typically, the same width, *w*_*n*_*= w* ≅ 2.0 kK, evaluated from the experimental absorption spectrum, was arbitrarily adopted for all involved bands. For the molar concentration, based on MC simulations of the Ru dye adsorbed layer, it was obtained *c* = 1.86 mol/dm^3^ for RuLp, *c* = 1.23 mol/dm^3^ for B1 and *c* = 1.31 mol/dm^3^ for B2, from average volumes occupied by the adsorbed molecules. One can see that the dye monolayer is rather compact and closely packed if one compares these values with the 2.0 M value for the RBY(PF_6_)_2_ crystal structure^43^ and the 2.4 M and 1.2 M values estimated earlier^16,18^ from absorption data for monoruthenium and biruthenium complexes, respectively, in the solid neat films.

## Electronic supplementary material

Below is the link to the electronic supplementary material.

Supplementary Material 1

## Data Availability

All the data are available on request from the corresponding author.
