# Supplementary material for: Excited states of mono- and biruthenium(II) complexes adsorbed on nanocrystalline titanium dioxide studied by electroabsorption spectroscopy
Source: Sci Rep. 2025 Feb 7;15:4562. doi: 10.1038/s41598-024-81957-z (PMC11803096; doi:10.1038/s41598-024-81957-z)
Supplement: Supplementary file 1 — Supplementary Material 1 [file 41598_2024_81957_MOESM1_ESM.docx]

SUPPLEMENTARY MATERIALS

Excited states of mono- and biruthenium(II) complexes adsorbed on nanocrystalline titanium dioxide studied by electroabsorption spectroscopy

Daniel Pelczarski^1*^, Błażej Gierczyk^2^, Maciej Zalas^2^, Malgorzata Makowska-Janusik^3^, Waldemar Stampor^1^

^1^ Department of Molecular Photophysics, Institute of Applied Physics and Mathematics, Gdańsk University of Technology, 11/12 Narutowicza str., 80-233 Gdańsk, Poland;

^2^ Faculty of Chemistry, Adam Mickiewicz University, Poznań, 8 Uniwersytetu Poznańskiego str., 61-614 Poznań, Poland;

^3^ Faculty of Science and Technology, Jan Dlugosz University, Al. Armii Krajowej 13/15, 42-200 Czestochowa, Poland;

**Table 1S.** Results of fitting model parameters for EA spectra of Ru(II) complexes. The uncertainty of parameters Δμ and Δ*p* is around 10%.

| **Ru complex** | **Band** |  | **Solid film** | | | | | **Adsorbed TiO_2_** | | | | | |
| --- | --- | --- | --- | --- | --- | --- | --- | --- | --- | --- | --- | --- | --- |
|  |  | **ABS** | **EA Model 1** | | **EA Model 2** | | | **ABS** | **EA Model 1** | | **EA Model 2** | | |
|  |  | **Posit.** | **Posit.** | ***f*·Δµ** | **Posit.** | ***f·*Δµ** | ***f*^2^·Δp** | **Posit.** | **Posit.** | ***f*·Δµ** | **Posit.** | ***f*·Δµ** | ***f*^2^·Δp** |
|  |  | **[kK]** | **[kK]** | **[D]** | **[kK]** | **[D]** | **[Å^3^]** | **[kK]** | **[kK]** | **[D]** | **[kK]** | **[D]** | **[Å^3^]** |
| **RuLp** | **1** | 18.85 | 19.12 | 5.5 (4.7) | 19.19 | 12 (9.8) | 70 | 18.97 | 19.07 | 4.9 (4.9) | 18.97 | 17 (17) | 140 |
|  | **2** | 21.04 | 21.17 | 3.8 (3.6) | 21.20 | 3.2 (3.1) |  | 21.03 | 20.90 | 10 (12) | 20.94 | 12 (15) |  |
|  | **3** | 22.28 | 22.14 | 16 (7.9) | 22.12 | 12 (6.1) |  | 22.28 | 22.13 | 17 (10) | 22.15 | 10 (6.2) |  |
|  | **4** | 23.19 | 23.37 | 8.0 (6.7) | 23.14 | 6.8 (5.5) |  | 23.18 | 23.41 | 12 (12) | 23.07 | 11 (12) |  |
|  | **5** | 25.03 | 24.81 | 13 (12) | 24.60 | 6.1 (5.6) |  | 25.02 | 24.65 | 6.6 (8.2) | 24.87 | 10 (12) |  |
| **B1** | **1** | 18.87 | 19.33 | 6.6 (5.1) | 19.31 | 12 (10) | 90 | 19.11 | 19.17 | 3.7 (3.7) | 19.02 | 24 (24) | 170 |
|  | **2** | 21.00 | 21.20 | 3.4 (3.2) | 21.26 | 2.9 (2.8) |  | 20.99 | 21.00 | 8.5 (10) | 21.02 | 13 (15) |  |
|  | **3** | 22.27 | 22.06 | 11 (5.1) | 22.12 | 12 (5.5) |  | 22.22 | 22.06 | 8.3 (6.4) | 22.20 | 7.7 (5.9) |  |
|  | **4** | 23.17 | 23.03 | 14 (11) | 23.16 | 8.0 (6.6) |  | 23.18 | 22.92 | 11 (11) | 23.14 | 11 (11) |  |
|  | **5** | 24.96 | 24.97 | 15 (13) | 24.80 | 7.5 (6.6) |  | 25.09 | 24.85 | 10 (13) | 24.95 | 8.2 (10) |  |
| **B2** | **1** | 18.88 | 19.32 | 6.0 (6.0) | 19.20 | 10 (10) | 90 | 19.11 | 19.31 | 2.9 (2.9) | 18.79 | 16 (16) | 130 |
|  | **2** | 20.86 | 21.22 | 2.8 (3.2) | 21.44 | 2.0 (2.4) |  | 20.93 | 20.88 | 9.8 (11) | 20.93 | 12 (13) |  |
|  | **3** | 22.10 | 22.03 | 9.8 (7.0) | 22.12 | 11 (7.8) |  | 22.12 | 21.95 | 11 (7.1) | 22.08 | 10 (6.5) |  |
|  | **4** | 23.13 | 23.16 | 15 (16) | 23.21 | 10 (11) |  | 23.10 | 22.88 | 12 (12) | 23.09 | 12 (12) |  |
|  | **5** | 25.03 | 25.15 | 14 (18) | 24.76 | 12 (15) |  | 25.09 | 24.68 | 11 (14) | 25.00 | 9.2 (12) |  |


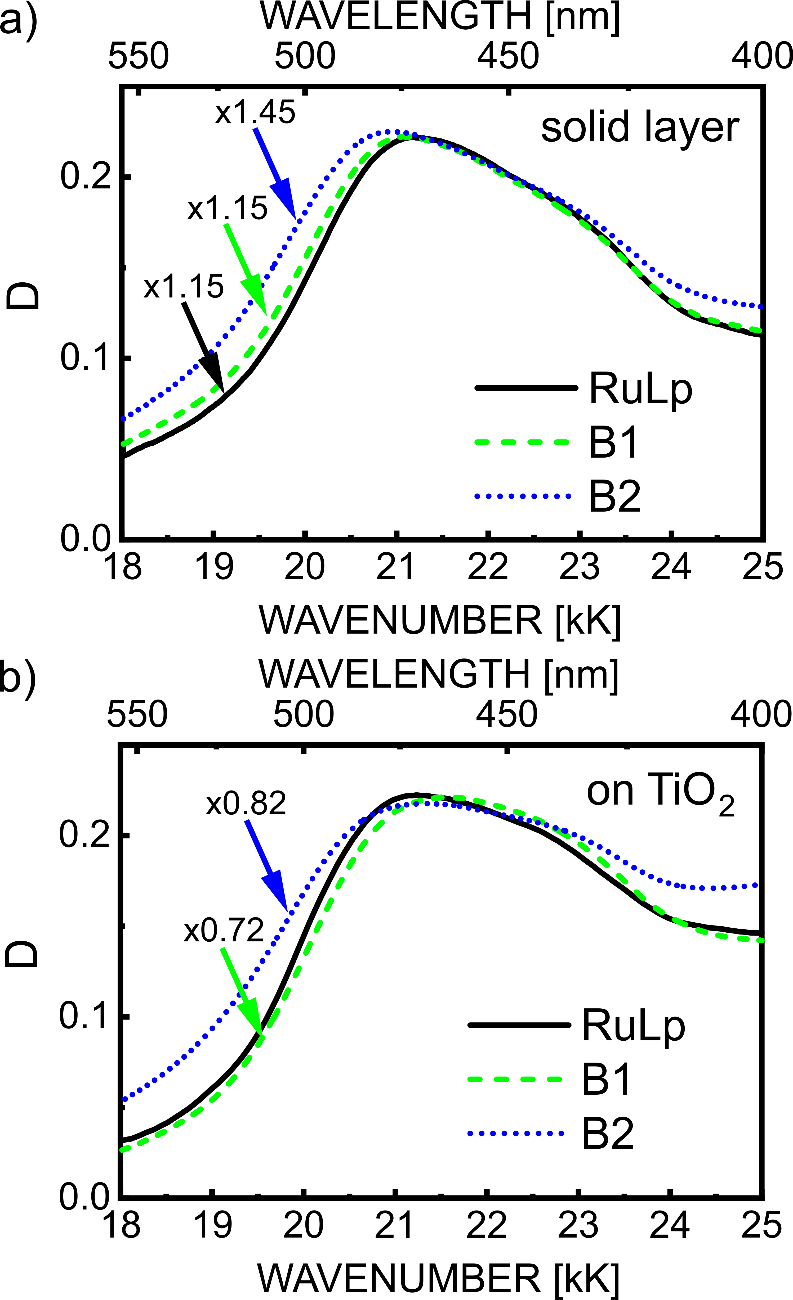


**Figure 1S.** Comparison of absorption spectra of the studied ruthenium complexes in the form of solid films (a) and adsorbed on TiO_2_ (b). Wavenumbers are expressed in kilokaysers, 1kK=1000 cm^-1^.
